# Farasan Island of Saudi Arabia confronts the measurable impacts of global warming in 45 years

**DOI:** 10.1038/s41598-022-18225-5

**Published:** 2022-08-22

**Authors:** Khaled Mohamed Khedher, Gasem Mohammad Abu-Taweel, Zarraq Al-Fifi, Mofareh D. Qoradi, Zainab Al-khafaji, Bijay Halder, Jatisankar Bandyopadhyay, Shamsuddin Shahid, LAATAR Essaied, Zaher Mundher Yaseen

**Affiliations:** 1 Department of Civil Engineering, College of Engineering, King Khalid University, Abha, 61421 Saudi Arabia; 2 Department of Civil Engineering, High Institute of Technological Studies, Mrezgua University Campus, 8000 Nabeul, Tunisia; 3 Department of Biology, College of Sciences, Jazan University, P.O. Box 2079, Jazan, 45142 Saudi Arabia; 4 Department of Geography, College of Arts, King Saud University, Riyadh, 11451 Saudi Arabia; 5 Building and Construction Engineering Technology Department, AL-Mustaqbal University College, Hillah, 51001 Iraq; 6 Department of Remote Sensing and GIS, Vidyasagar University, Midnapore, West Bengal India; 7 School of Civil Engineering, Faculty of Engineering, Universiti Teknologi Malaysia (UTM), 81310 Johor Bahru, Malaysia; 8 Company of Phosphate of Gafsa and Chemical Group of Tunisia, Appt. D6 Immeuble Ramsis Avenue Habib Bourguiba, 2034 Ezzahra Ben Arous, Tunisia; 9 New Era and Development in Civil Engineering Research Group, Scientific Research Center, Al-Ayen University, Thi-Qar, 64001 Iraq

**Keywords:** Physical oceanography, Hydrology, Climate and Earth system modelling, Climate-change impacts

## Abstract

Coastal vulnerability assessment is the key to coastal management and sustainable development. Sea level rise (SLR) and anthropogenic activities have triggered more extreme climatic events and made the coastal region vulnerable in recent decades. Many parts of the world also noticed increased sediment deposition, tidal effects, and changes in the shoreline. Farasan Island, located in the south-eastern part of Saudi Arabia, experienced changes in sediment deposition from the Red Sea in recent years. This study used Digital Shoreline Analysis System (DSAS) to delineate the shoreline changes of Farasan Island during 1975–2020. Multi-temporal Landsat data and DSAS were used for shoreline calculation based on endpoint rate (EPR) and linear regression. Results revealed an increase in vegetation area on the island by 17.18 km^2^ during 1975–1989 and then a decrease by 69.85 km^2^ during 1990–2020. The built-up land increased by 5.69 km^2^ over the study period to accommodate the population growth. The annual temperature showed an increase at a rate of 0.196 °C/year. The sea-level rise caused a shift in the island's shoreline and caused a reduction of land by 80.86 km^2^ during 1975–2020. The highly influenced areas by the environmental changes were the north, central, northwest, southwest, and northeast parts of the island. Urban expansion and sea-level rise gradually influence the island ecosystem, which needs proper attention, management, policies, and awareness planning to protect the environment of Farasan Island. Also, the study’s findings could help develop new strategies and plan climate change adaptation.

## Introduction

The shoreline, the common line between land and water^1^, is significantly affected by land use and environmental changes^2–4^. Shoreline changes have been reported in different parts of the globe in recent years with the changes in the global environment^5,6^. Shoreline change due to global sea-level rise and environmental disturbances is the major challenge in coastal management. The tectonic and thermal effects are the main reason for shoreline shifting. The coastal erosion and accretion also caused the shoreline to shift to the land and ocean. Global ice melting, one key factor for sea-level rise (SLR), also caused shoreline change and coastal vulnerability. Changes in sedimentation dynamics due to the tidal effect are the major cause of erosion and accretion^7,8^. Besides, SLR is a major controlling factor of shoreline changes^9^. The shoreline change assessment is vital for understanding the erosion-accretion rate, environmental conditions, biodiversity-related conditions, future shoreline change prediction, safe navigation, hazard mapping, and coastal resource management.

Many methods have been used to detect shorelines. Shoreline mapping through field surveys provides the highest accuracy. However, it is time-consuming, costly and labour intensive^10,11^. Aerial photography and remote sensing technologies are widely used as an alternative to field surveys for mapping shorelines^12,13^. Different techniques have been proposed for delineating the shoreline changes using remote sensing techniques, like Light Detection and Ranging (LiDAR)^14,15^, Synthetic Aperture Radar (SAR) image^16^, the Video system^17^, and satellite images^18,19^. The multi-temporal satellite imagery among them is more extensively used to delineate the shoreline change^20,21^. Studies reported higher accuracy using multi-temporal satellite images because infrared bands indicate the land–water interface and facilitate better shoreline delineation in the coastal region^22,23^.

Land transformation due to global climate change and sea-level rise has caused an increase in coastal vulnerability^24^. Land area monitoring is useful for planning and managing anthropogenic and environmental activities on the earth's surface^25^. Global climate change and anthropogenic activities trigger thermal variation in the earth's surface and a gradual increase in the earth's temperature. The Land surface temperature (LST) can be used for monitoring environmental problems, surface temperature variation and heat stress^25,26^. The LST and vegetation in a scatter plot reveal pixels' chronological trajectory ranging from low-temperature high vegetation conditions to identifying high-temperature low vegetation^27,28^. The geospatial indices also help monitor the vegetation and built-up area condition and expansion of the built-up land, vegetation and other LU/LC on the earth's surface. Therefore, many geospatial indices have been used to map, monitor, and analyze an area's vegetation and built-up scenarios.

Erosion control and coastal zone management are critical aspects of extreme environmental events. Endpoint rate (EPR), Linear Regression (LR) and Average Rate (AOR) are used for shoreline change detection and shift identification. EPR model can predict future shorelines based on historical shoreline change analysis, while the LR can predict the short-term shoreline change^29^. Integrating those methods with remote sensing and GIS techniques can provide a semi-automated shoreline change analysis system identifying the coastal and geomorphological dynamics.

The Farasan Island is located in the south-eastern part of Saudi Arabia and near the Jazan Province. The Farasan Island Marine Sanctuary (FIMS) is one of the important parts of Saudi Arabia. Some inhabited areas are also located on this island, like Sair, Khutob, Al Sageed, Abu Twoq, Farasan Al Meharrq, Al Qessar and Qummah. Its location in the southern part of the Red Sea and aerial transportation increased the habitation in recent years. Due to sea-level rise (SLR), the water level of the Red Sea has increased, and the area of Farasan Island changed significantly. Sedimentation caused a build-up of the land, while sea-level rise (SLR) caused a negative shoreline change, triggering the land transformation on Farasan Island. The shoreline change influences local environmental conditions and natural biodiversity-related problems. Sediment deposition affected Farasan Island's biodiversity, including mangroves, vegetation, grassland, and shallow water. Shoreline shift also affected human habitation on the island. Research also indicates that the shell middens distribution of Farasan Island is influenced by shoreline shift^30^.

Based on the exhibited literature review and the significance of the research case study focus, the current research was motivated to investigate the geological and geographical characteristics of Farasan Island. The major objectives of this study are (1) shoreline analysis using Landsat Data, (2) calculation of shoreline change rate using the Digital Shoreline Analysis System (DSAS), (3) calculation of the island area for different study years, (4) land use and land cover analysis of the Farasan Island for different years, (5) LST and geospatial indices-based. The shoreline over the past five decades (1975–2020) was inspected and studied comprehensively using multi-temporal satellite images. In addition, several other geospatial indices, like the Normalized Difference Vegetation Index (NDVI), and Normalized Difference Built-up Index (NDBI), were examined for a better understanding of the island's geographical changes. The feasibility of GIS integrated with the emerging satellite dataset was examined for the shoreline modelling. Landsat Multispectral Scanner (MSS), Thematic Mapper (TM) and Operational Land Imager/Thermal Infrared Sensor (OLI/TIRS) data were used in delineating the shoreline change. The attained modelling results are expected to debate and discuss the historical shoreline changes of Farasan Island, providing a more informative hydrological, environmental and geographical understanding. This study may be helpful for the local policymakers, researchers, planners, administrators and other stakeholders for the sustainable development of Farasan Island.

## Study area

**Figure 1:**
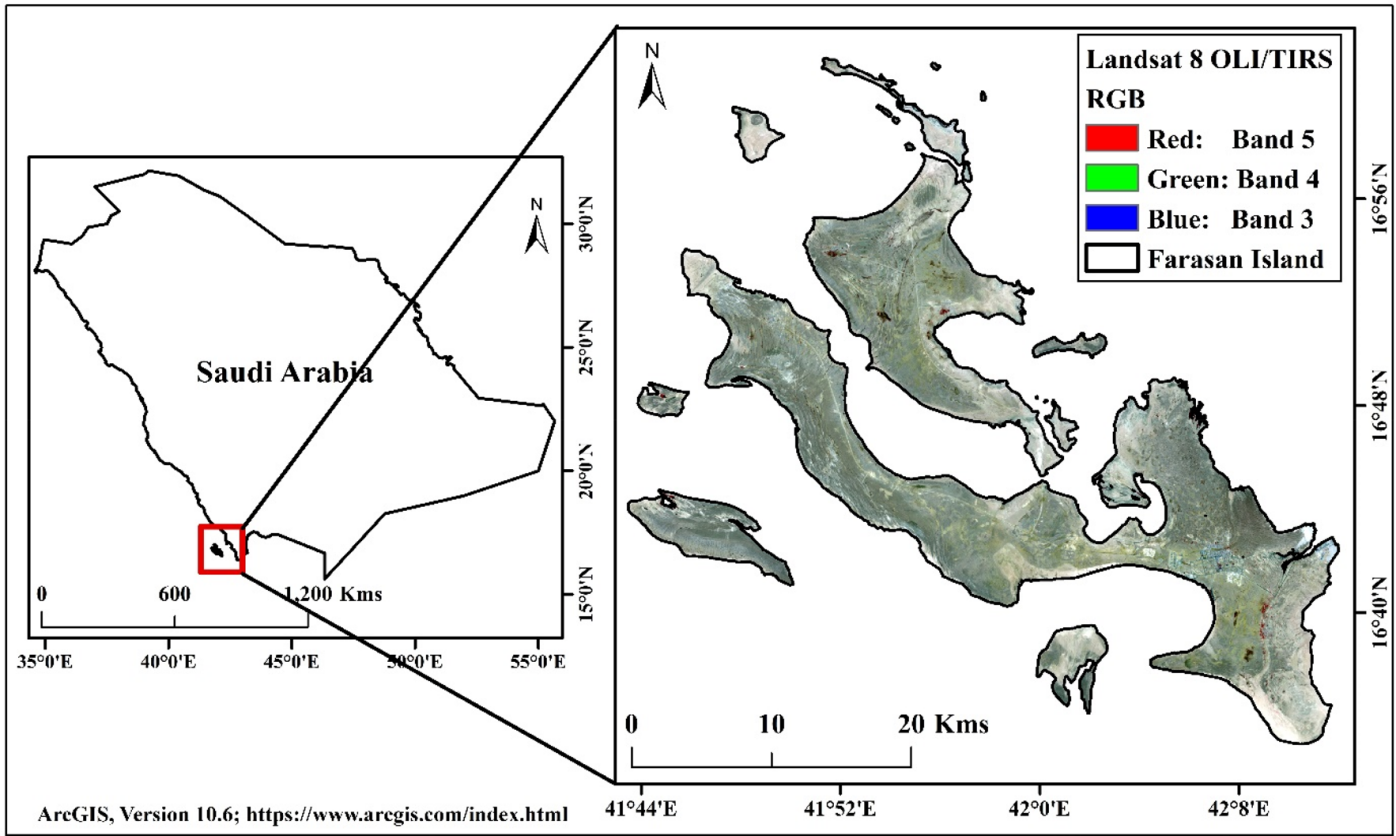
The location map of the studied Farasan Islands, Saudi Arabia^31,32^. Saudi Arabia boundary data was downloaded from DIVA-GIS website (https://www.diva-gis.org/). The satellite data was downloaded from USGS earth explorer (https://earthexplorer.usgs.gov/). The map was generated using ArcGIS software, version 10.6 (https://support.esri.com/zh-cn/products/desktop/arcgis-desktop/arcmap/10-6-1).

The Farasan Island is located in the southern parts of the Red Sea, approximately 40 km from Jazan, Saudi Arabia. Geographically, the area is bounded between 16° 8″–17° 10″ N and 41° 22″–42° 0″ E (Fig. 1, https://www.diva-gis.org/gdata)^31,32^. April to October is the summer season, and November to March is the low hot season in the area. The annual average temperature of the island is 30 °C. The relative humidity is 70–80% in the winter and 65–75% in summer. The most rainfall in the region occurs in April. The Farasan Island is a group of 176 islands in the Red Sea. The islands are inhabited by 20,000 population and belong to Jazan Province (Report on 2010). The building contraction and tourist attraction are hammering the local climate. Most of the built-up lands are gradually increasing due to habited suitability of this location and tourist attraction of both national and international. The Farasan is the largest city on this Island. Besides, some cities are noticeably changing the built-up expansion on the islands (Fig. 1). The daytime highest temperature is around 40–44 °C in the summer while the nighttime temperature reduces to 2–3 °C. The wind speed is around 21–25 km/hr, which varies from coast to inland. Most of the Island is covered by barren land where some vegetation and built-up land have recently been noticed.

## Materials and methods

### Data used

**Figure 2:**
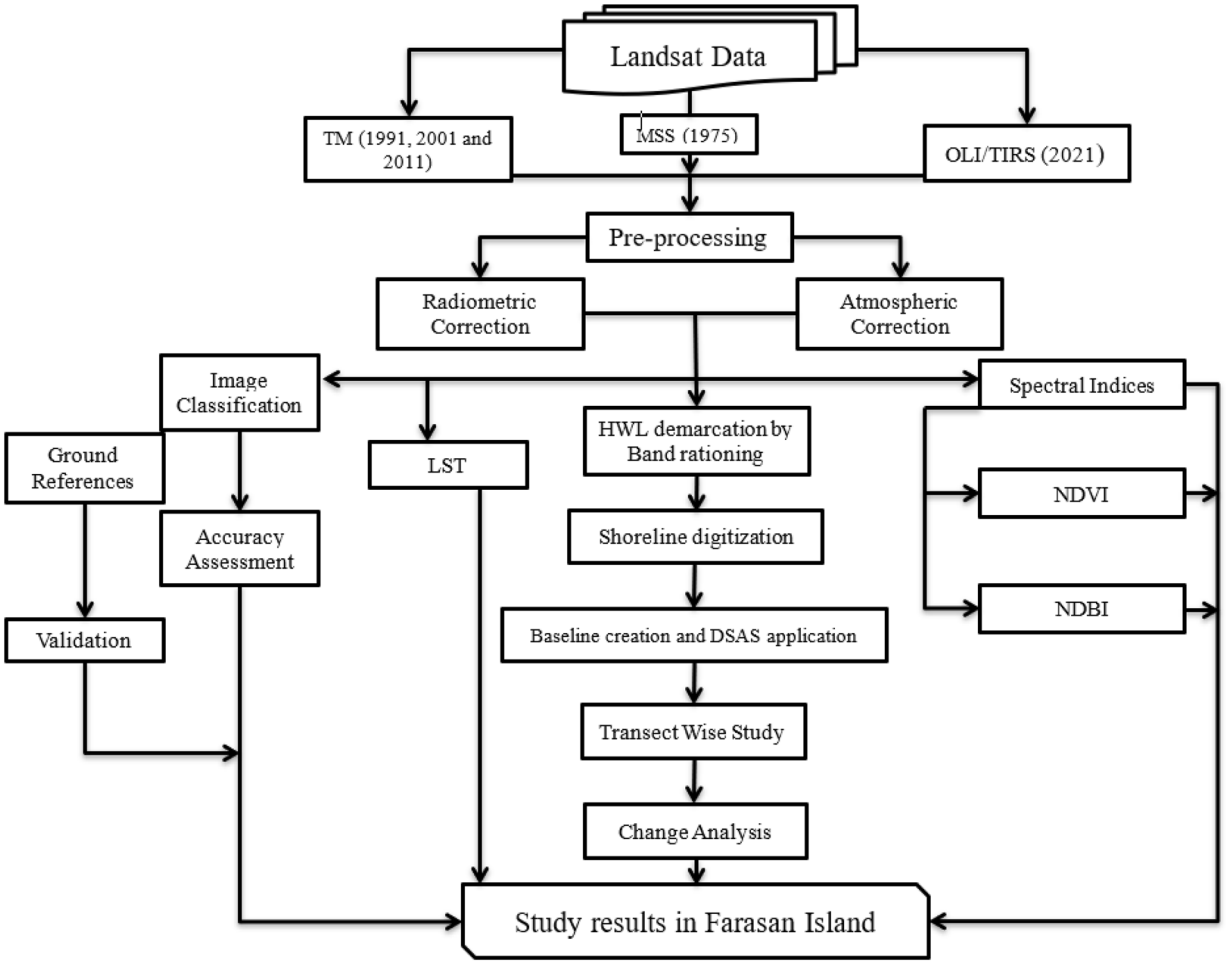
The adopted methodological framework of the current study.

Remote sensing-based satellite imageries were used for land use and land cover classification, thermal variation and geospatial indices estimation, and shoreline change analysis. Landsat (MSS), Landsat 5 (TM) and Landsat 8 (OLI/TIRS) data were used for estimating the shoreline of different years and calculating the yearly change rate of shoreline and LU/LC. Geospatial indices and LST were estimated using four satellite imageries with ten-year intervals (1990–2020). The Landsat imageries of 1975, 1990, 2000, 2010 and 2020 were used for shoreline shifting analysis (Table 1). The Landsat data was downloaded from the US Geological Survey (USGS) Earth Explorer website (http://www.earthexplorer.usgs.gov). The data was downloaded from the USGS website with less than 10% cloud cover. The medium-resolution Landsat imageries were widely used for land-related studies and shoreline sifting analysis in the coastal regions (Fig. 2).

**Table 1:** Satellite image used for the studied region.

Satellite	Sensor	Date	Path and row	Data source
Landsat 2	MSS	22-06-1975	180, 048	https://earthexplorer.usgs.gov/
Landsat 5	TM	15-04-1990	167, 048	https://earthexplorer.usgs.gov/
		10-04-2000	167, 048	https://earthexplorer.usgs.gov/
		01-02-2010	167, 048	https://earthexplorer.usgs.gov/
Landsat 8	OLI/TIRS	01-04-2020	167, 048	https://earthexplorer.usgs.gov/

### Image pre-processing

The shoreline shift analysis system needs geometric, atmospheric and radiometric correction of the satellite image^33^. The pixel matching is the main condition for pre-processing; otherwise, the change cannot be estimated from the reflectance values^34^. The radiometric correction may include subtraction of atmospheric correction (FLAASH method), view angles and terrain correction, reduction of the calibration and sensor calibration^35^. The atmospheric correction is essential for wavelength-related information. The FLAASH model was used for atmospheric correction, which modifies pixel-based X corresponding to the solar wavelength range^36,37^. The model was used for aerosol/haze removal and clarity of the Landsat imageries. After radiometric correction, Landsat imageries were geometrically corrected. The imageries were included in the World Geodetic System (WGS 1984) datum and Universal Transverse Mercator (UTM) projection system. The tidal information of Farasan Island is around 1.2 m at 4.40 a.m. (high tide) and 0.6 m at 11.02 a.m. (low tide) (https://www.tideschart.com/Saudi-Arabia/Jazan-Region/Farasan/). The tidal data is not shown in the manuscript because this study used satellite-based high water level data, which also considers the tidal effects.

### Image classification and post-classification

LU/LC classification is the key monitoring system for identifying human intervention, extreme environmental conditions and important aspects of earth surface phenomenon^38^. Anthropogenic activities, global climate change, and sea-level rise have made the coastal land of the Farasan Island dynamics. The supervised classification technique with a maximum likelihood algorithm was used for land use/land cover classification^39,40^. ArcGIS 10.6 was used for Landsat data classification for different years like 1990, 2000, 2010 and 2020. Due to image unavailability and bad image quality, the data for the year 1975 was not classified.

The accuracy assessment and kappa coefficient identification of each classified imageries are more useful for monitoring the error matrix for each classified year^41^. The ERDAS Imagine software was used for generating the accuracy assessment and kappa coefficient (Table 2). The field data and Google Earth data were used for validation and ground referencing. The accuracy assessment and kappa coefficient were calculated using formulas given in Eqs. (1) and (2)

1 $$OA=\left(\frac{{{\sum }_{i=1}^{k}}{{n}_{ij}}}{n}\right)$$

2 $${K}_{i}=\frac{(\text{Observed accuracy}-\text{Chance accuracy})}{(1 -\text{ Chance accuracy})}$$

where *n*_*ij*_ represents the diagonal elements in the error matrix, k is the total number of classes in the land use/land cover classification, n is the total number of samples in the error matrix, and *K*_*i*_ represents the kappa coefficient.

**Table 2:** Kappa coefficient scale.

Sl no.	Value of K	Strength of agreement
1	< 0.20	Poor
2	0.21–0.40	Fair
3	0.41–0.60	Moderate
4	0.61–0.80	Good
5	0.81–1.00	Very good

### Land surface temperature

#### LST for landsat TM data

The LST was calculated from the brightness temperature of Landsat TM images in a two-stop process^42^. The procedure is as follows.

Conversion of the digital number (DN) to spectral radiance (L) (USGS 2001) by Eq. (3)

3 $$L=\left(\frac{{L}_{\text{max}-{L}_{min}}}{{DN}_{max}}\right)\times Band+{L}_{min}$$

where L is the spectral radiance, L_min_ is 1.238 (Spectral radiance of the DN value 1), L_max_ is 15.6000 (Spectral radiance of DN value 255), and DN is the digital number.

Conversion of spectral radiance to temperature in kelvin (USGS 2001) is calculated by Eq. (4)

4 $$Tb=\frac{K2}{(\frac{K1}{L\lambda }+1)}$$

where K1 is the calibration constant 1 (607.76), K2 is the calibration constant 2 (1260.56), and Tb is the surface temperature (kelvin).

Calculation of NDVI

5 $$NDVI=\frac{(NIR-R)}{(\text{NIR}+\text{R})}$$

The NDVI value ranges between − 1 and + 1. The 0 to + 1 represents the vegetation, and the negative values represent other LU/LC classes.

Land surface emissivity (LSE) was calculated based on NDVI. It uses the NDVI Thresholds Method, NDVITHM, by applying Eq. (6)^43^.

6 $$LSE = \left( {1.0094 + 0.047} \right) \times In\;\left( {NDVI} \right)$$

The NDVI value ranges from 0.157 to 0.727. The corresponding input LST constant values are used when the NDVI values are out of the range (0.157–0.727).

Conversion of Kelvin to Celsius^44^ is estimated by Eq. (7).

7 $$LST = Tb - 273.15$$

#### LST for landsat OLI/TIRS data

The proportion of vegetation is calculated by minimum, and maximum NDVI values^45^ using Eq. (8).

8 $${\text{Pv}} = \left( {\frac{{NDVI{-}NDVI_{\min } }}{{NDVI_{\max } {-}NDVI_{\min } }}} \right)^{2}$$

Land surface emissivity (LSE) was calculated from *P*_*v*_ value. It uses the NDVI Thresholds method by applying Eq. (9)^46,47^.

9 $$LSE=0.004\times Pv+0.986$$

Conversion of Kelvin to Celsius^47,48^ is estimated using Eq. (10):

10 $$LST=\frac{BT}{\{1+\left[\frac{{\uplambda {\text{BT}}}}{\uprho }\right]\text{In}(\text{LSE})}$$

### Geospatial indices

#### NDVI

The vegetation index is more useful for monitoring the vegetation condition and change analysis of vegetation area. The Landsat TM and OLI/TIRS data (1990–2020) were used for calculating the vegetation index (NDVI). The vegetation-covered and degradation areas were also identified using NDVI (Eq. 5).

### NDBI

The Normalized Difference Built-up Index (NDBI) was calculated for the urban area using Landsat TM and OLI/TIRS data. The shortwave infrared (SWIR) is characteristically higher reflectance compared to the near-infrared region. This built-up index (Eq. 11) is used for built-up area and land use planning^49^:

11 $$NDBI=\frac{(NIR-SWIR)}{(\text{NIR}+\text{SWIR})}$$

*NDBI* value ranges between − 1.0 and + 1.0. The positive values of NDBI were considered as the build-up area.

### Shoreline extraction technique

Several automatic and semi-automatic techniques have been recently used to extract shorelines from optical satellite imagery. Supervised and unsupervised classifications^20,50–52^, band rationing^22,53^, and threshold values^54,55^ are among the most well-known and straightforward methods. The High Water Line (HWL) is generally used to delineate the shoreline shifting from different years’ satellite Landsat datasets. HWL is also often used as the identifier^56^ for the highest point of the earlier high tide derived from remote sensing data and the coast by a perceptible wet/dry strip^1^. The HWL was estimated using band rationing and then digitized in ArcGIS 10.6 software. The employed band rationing of Landsat TM was B5/B2, and Landsat OLI/TIRS was B6/B3. The reclassifications of the calculated bands were used for shoreline change analysis. The values less than 1 denote water pixels, and 0 denotes the land pixels. After reclassification, the raster-to-vector conversion and line smoothing technique were used for shoreline extraction.

### Baseline creation and laying of transects

After digitizing the shoreline of Farasan Island for different years from 1975 to 2020, all vector lines were clipped to generate a common shapefile. The buffer was created to identify the baseline for calculating the shoreline shifting rate over the period. Transects were created at 500 m intervals for the entire island.

### Uncertainty in shoreline shifting

The error in shoreline change analysis was identified using the Landsat dataset^57^ and the shoreline shifting statistical significance analysis^58^. Test and train data were used to assess shoreline shifting data, where the test data was field datasets or ground point data. The ground point data were used to generate the error matrix and get the high accuracy of shoreline change analysis.

### Transect-from-baseline approach

The Digital Shoreline Analysis System (DSAS) tool derived from the USGS website was used to delineate transect and shoreline change analysis. ArcGIS 10.6 was used to create the transect line for calculating the shoreline shifting each year. The EPR and LRR models were used to analysis of shoreline position changes^59,60^. The DSAS tool^61^ was used for this purpose. The EPR and LRR models were used for shoreline change analysis and transect-based land alteration analysis.

The End Point Rate (EPR) is calculated by the distance of the total shoreline shift in the different periods between each transect's initial and newest measurement. The equation of EPR calculation is:

12 $$EPR=\left(\frac{A-B}{T}\right)$$

where, A − B represents the distance of the shoreline in meters and T represents the time between the youngest and oldest shoreline area.

The endpoint rate was calculated for each data pair, like 1975–1990, 1990–2000, 2000–2010 and 2010–2020. The rate was calculated using the distance between two shoreline areas for different intervals^42,77^. The linear regression rate was used to calculate the rate of the entire study area (1975–2020). The LRR method was used to fit the least-squares lines to all shoreline points^63^. The different 4 years of data were used to map and monitor the shoreline change and measure the shoreline change rate.

## Application results

### Land transformation

Land transformation is the key research topic for monitoring land degradation and earth surface phenomena study. Global warming, climate change, extreme environmental events and urban expansion influence the land transformation worldwide, triggering land losses, vegetation degradation, built-up expansion, infrastructural development, increased heat stress and air pollution. The land transformation study of Farasan Island was necessary to generate land use and land cover-related information and land loss area identification. The global sea-level rise and climate change have increased the vulnerability of shorelines on the Farasan Island^31,64^, figures were generated using ArcGIS software 10.6, https://www.esri.com/en-us/arcgis/about-arcgis/overview.

**Figure 3:**
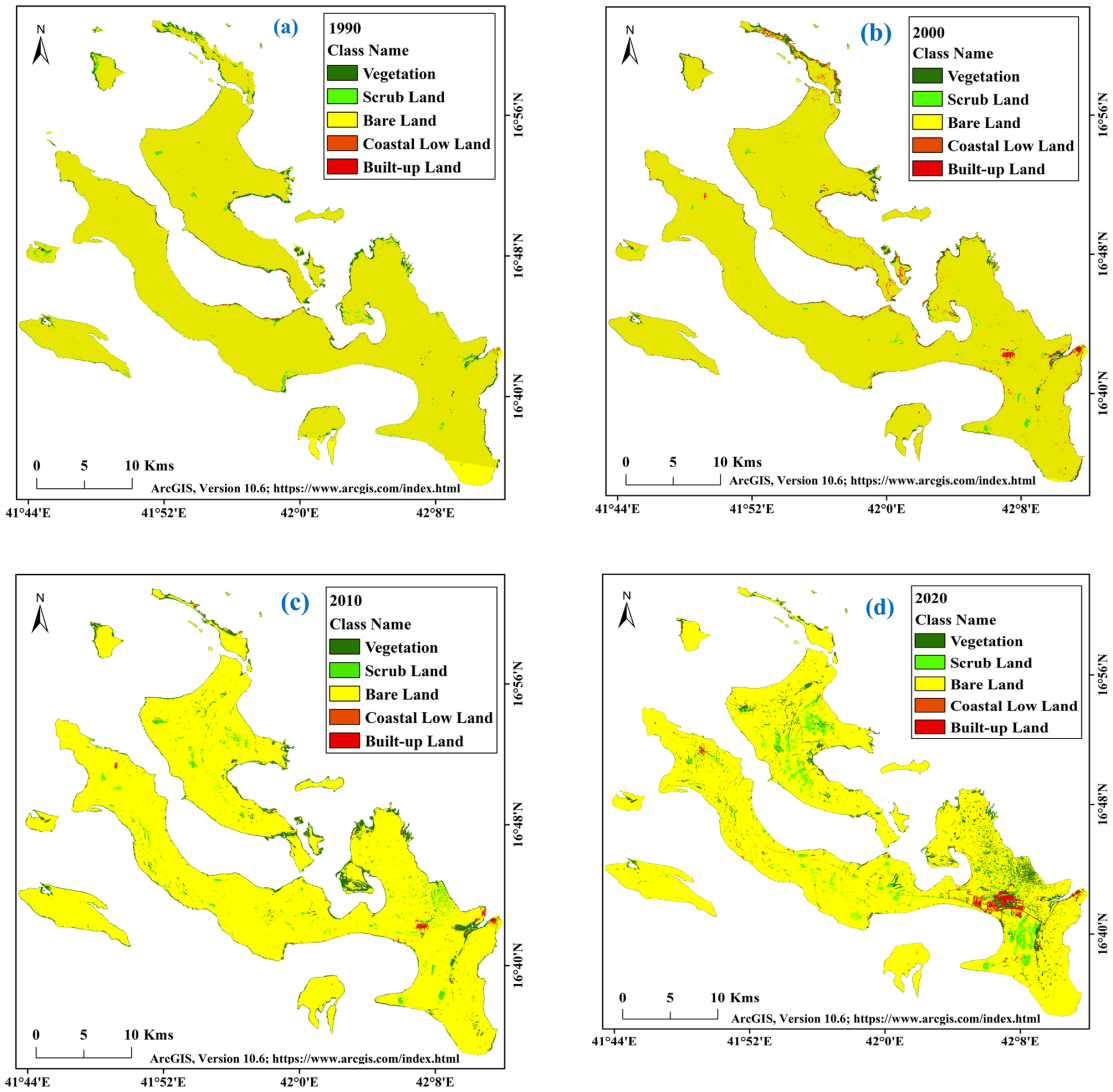
Land use and land cover classification maps of Farasan Islands; (**a**) 1990; (**b**) 2000; (**c**) 2010; (**d**) 2020^31,64^. The satellite datasets were downloaded from USGS earth explorer (https://earthexplorer.usgs.gov/). The map was generated using ArcGIS software, version 10.6 (https://support.esri.com/zh-cn/products/desktop/arcgis-desktop/arcmap/10-6-1).

Land use and land cover of Farasan Island is mostly affected by the extreme natural environmental condition and sea-level rise. The total area of Farasan Island has been reducing due to sea-level rise and shoreline shifting. Most of the areas are covered by bare land and scrubland, as identified in the study area. Vegetation is the most dominating factor in developing a healthy environment and increasing the air quality in an area. The development of urban expansion causes increased heat stress, oxygen deficiency, land transformation and ecological disturbances. Five types of LU/LC classes were identified in Farasan Island; vegetation, scrub Land, bare land, coastal lowland and built-up land (Fig. 3a–d). The results revealed an increase in vegetation area over the study period. The notified area of vegetation in different years was 22.74 km^2^ (1990), 25.39 km^2^ (2000), 35.84 km^2^ (2010), and 39.92 km^2^ (2020). The vegetation areas were mostly mangroves. Besides, some densely vegetated land was identified in the coastal and some built-up locations. Table 3 shows the areas that belong to different land use and the percentage of each classified area to total area. The scrublands, the dominant land use in the area, showed a gradual increase over time. The area of scrubland in different years were 4.93 km^2^ (1990), 8.24 km^2^ (2000), 11.25 km^2^ (2010), and 23.50 km^2^ (2020) and the bare lands were 612.23 km^2^ (1990), 597.30 km^2^ (2000), 563.47 km^2^ (2010), and 542.38 km^2^ (2020) (Table 3).

**Table 3:** Area calculation of different LU/LC classes of Farasan Island.

Class name	Area (sq. km)				Area (%)			
	1990	2000	2010	2020	1990	2000	2010	2020
Vegetation	22.74	25.39	35.84	39.92	3.52	3.99	5.80	6.50
Scrub land	4.93	8.24	11.25	23.50	0.76	1.30	1.82	3.83
Bare land	612.23	597.30	563.47	542.38	94.81	94.01	91.19	88.31
Coastal low land	3.69	1.16	1.64	0.52	0.57	0.18	0.27	0.08
Built-up land	2.16	3.25	5.73	7.85	0.33	0.51	0.93	1.28
Total area	645.75	635.34	617.93	614.17				

**Figure 4:**
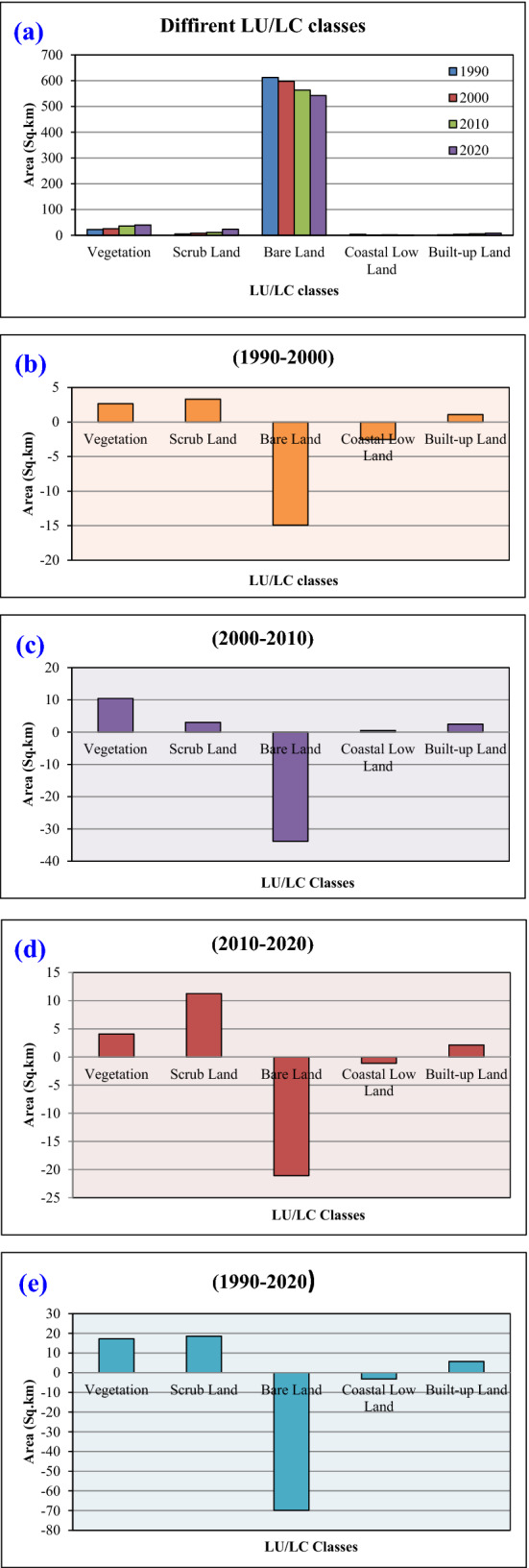
Area of different LU/LC classes; (**a**) LU/LC area for different years; (**b**) area change during 1990–2000; (**c**) area change during 2000–2010; (**d**) area change during 2010–2020; (**e**) area change during 1990–2020.

Coastal lowland is mainly located in the shoreline or coastline area. A significant portion of the study area is the coastal lowland. Its coverage in different years were as follows: 3.69 km^2^ (1990), 1.16 km^2^ (2000), 1.64 km^2^ (2010), and 0.52 km^2^ (2020) respectively (Fig. 4a–e). The coastal lowland gradually decreased due to global climate change-induced sea-level rise. The built-up land increased due to population pressure and infrastructural development. Farasan, Sair, Abu Twoq, Khutob, Qummah, Al Qessar and Al Meharrg are the places where the built-up land has increased simultaneously. Besides, there are many vegetated lands like Al Meharrq, Qandal Forest, Khutob and the northern coastal side in this study area.

Table 4 was used to calculate the areal change in land use in different periods on Farasan Island. The vegetation, scrubland, and built-up lands showed an increase, while bare land and coastal lowland showed a decrease due to shoreline shifting, urban expansion and population pressure. The changes in vegetation cover were 2.65 km^2^ (1990–2000), 10.45 km^2^ (2000–2010), 4.08 km^2^ (2010–2020), and 17.18 km^2^ (1990–2020). The vegetation areas were mostly increased in the south, north and north-eastern parts of the island. Scrubland and built-up land were also increased in the study area by 18.57 km^2^ and 5.69 km^2^, respectively, during 1990–2020. Bare land was decreased by 14.93 km^2^ (1990–2000), 33.83 km^2^ (2000–2010), 21.09 km^2^ (2010–2020), and 69.85 km^2^ (1990–2020). The accuracy and kappa coefficient of those classification maps were within the acceptable limits. The accuracy assessments of different years were 87.33% (1990), 85.28% (2000), 85.84% (2010), and 87.39% (2020). The kappa coefficient were 0.81 (1990), 0.79 (2000), 0.80 (2010), 0.83 (2020) respectively (Tables 5, 6, 7, 8). The LU/LC maps revealed the changes in shoreline area identified in the classification and shoreline shifting maps. The LU/LC alteration is also an influencing factor for thermal variation and vegetation degradation, where the main city of Farasan gradually noticed high built-up expansion and transportation development. The infrastructural development caused increased pollution, land production losses, climate change, and many more. They also triggered land alteration and thermal variation. Therefore LU/LC study and thermal variation investigation are important to protect the environment using novel technologies and adaptation policies.

**Table 4:** Changes in the area of Farasan Island in different periods.

Class name	Area changes in different years (sq. km)			
	(1990–2000)	(2000–2010)	(2010–2020)	(1990–2020)
Vegetation	2.65	10.45	4.08	17.18
Scrub land	3.31	3.01	11.25	18.57
Bare land	− 14.93	− 33.83	− 21.09	− 69.85
Coastal low land	− 2.53	0.48	− 1.12	− 3.17
Built-up land	1.09	2.48	2.12	5.69

**Table 5:** Accuracy assessment and kappa coefficient for the year of 1990.

Class name	Ground truth/references					Row total	Commission error	User accuracy
	Vegetation	Scrub land	Bare land	Coastal low land	Built-up land			
Vegetation	**29**	4	3	1	1	38	23.68%	76.32%
Scrub land	5	**48**	2	1	3	59	18.64%	81.36%
Bare land	4	7	**149**	2	1	163	8.59%	91.41%
Coastal low land	0	1	1	**15**	0	17	11.76%	88.24%
Built-up land	0	1	1	0	**21**	23	8.70%	91.30%
Column total	38	61	156	19	26	**300**		
Omission error	23.68%	21.31%	4.49%	21.05%	19.23%			
Producer accuracy	76.32%	78.69%	95.51%	78.95%	80.77%			
Overall accuracy	**87.33%**			Kappa Coefficient	**0.81**			

The [bold] value is necessary to identify the accuracy assessment and kappa coefficient.

**Table 6:** Accuracy assessment and kappa coefficient for the year of 2000.

Class name	Ground truth/references					Row total	Commission error	User accuracy
	Vegetation	Scrub land	Bare land	Coastal low land	Built-up land			
Vegetation	**35**	5	2	2	1	45	22.22%	77.78%
Scrub land	6	**57**	3	2	2	70	18.57%	81.43%
Bare land	7	5	**137**	1	3	153	10.46%	89.54%
Coastal low land	2	3	1	**21**	1	28	25.00%	75.00%
Built-up land	0	1	1	0	**28**	30	6.67%	93.33%
Column total	50	71	144	26	35	**326**		
Omission error	30.00%	19.72%	4.86%	19.23%	20.00%			
Producer accuracy	70.00%	80.28%	95.14%	80.77%	80.00%			
Overall accuracy	**85.28%**			Kappa coefficient	**0.79**			

The [bold] value is necessary to identify the accuracy assessment and kappa coefficient.

**Table 7:** Accuracy assessment and kappa coefficient for the year of 2010.

Class name	Ground truth/ references					Row total	Commission error	User accuracy
	Vegetation	Scrub land	Bare land	Coastal low land	Built-up land			
Vegetation	**41**	6	2	1	2	52	21.15%	78.85%
Scrub land	6	**67**	4	2	3	82	18.29%	81.71%
Bare land	8	4	**134**	0	2	148	9.46%	90.54%
Coastal low land	2	1	1	**14**	1	19	26.32%	73.68%
Built-up land	1	1	1	0	**35**	38	7.89%	92.11%
Column total	58	79	142	17	43	**339**		
Omission error	29.31%	15.19%	5.63%	17.65%	18.60%			
Producer accuracy	70.69%	84.81%	94.37%	82.35%	81.40%			
Overall accuracy	**85.84%**			Kappa coefficient	**0.80**			

The [bold] value is necessary to identify the accuracy assessment and kappa coefficient.

**Table 8:** Accuracy assessment and kappa coefficient for the year of 2020.

Class name	Ground truth/references					Row total	Commission error	User accuracy
	Vegetation	Scrub land	Bare land	Coastal low land	Built-up land			
Vegetation	**49**	5	3	1	1	59	16.95%	83.05%
Scrub land	7	**71**	3	1	4	86	17.44%	82.56%
Bare land	4	3	**124**	0	2	133	6.77%	93.23%
Coastal low land	2	0	1	**11**	1	15	26.67%	73.33%
Built-up land	2	1	2	0	**43**	48	10.42%	89.58%
Column total	64	80	133	13	51	**341**		
Omission error	23.44%	11.25%	6.77%	15.38%	15.69%			
Producer accuracy	76.56%	88.75%	93.23%	84.62%	84.31%			
Overall accuracy	**87.39%**			Kappa coefficient	**0.83**			

The [bold] value is necessary to identify the accuracy assessment and kappa coefficient.

### Topographical distribution of LST

Temperature variation influences the heat stress, thermal variation, urban thermal field variation, sea surface temperature (SST) and urban heat island effect. Most of the world's population live in coastal area, and the temperature in many coastal regions has increased recently. Temperature variation is the key metric of climate change in arid and semi-arid regions. Saudi Arabia is significantly affected by the temperature increase.

**Figure 5:**
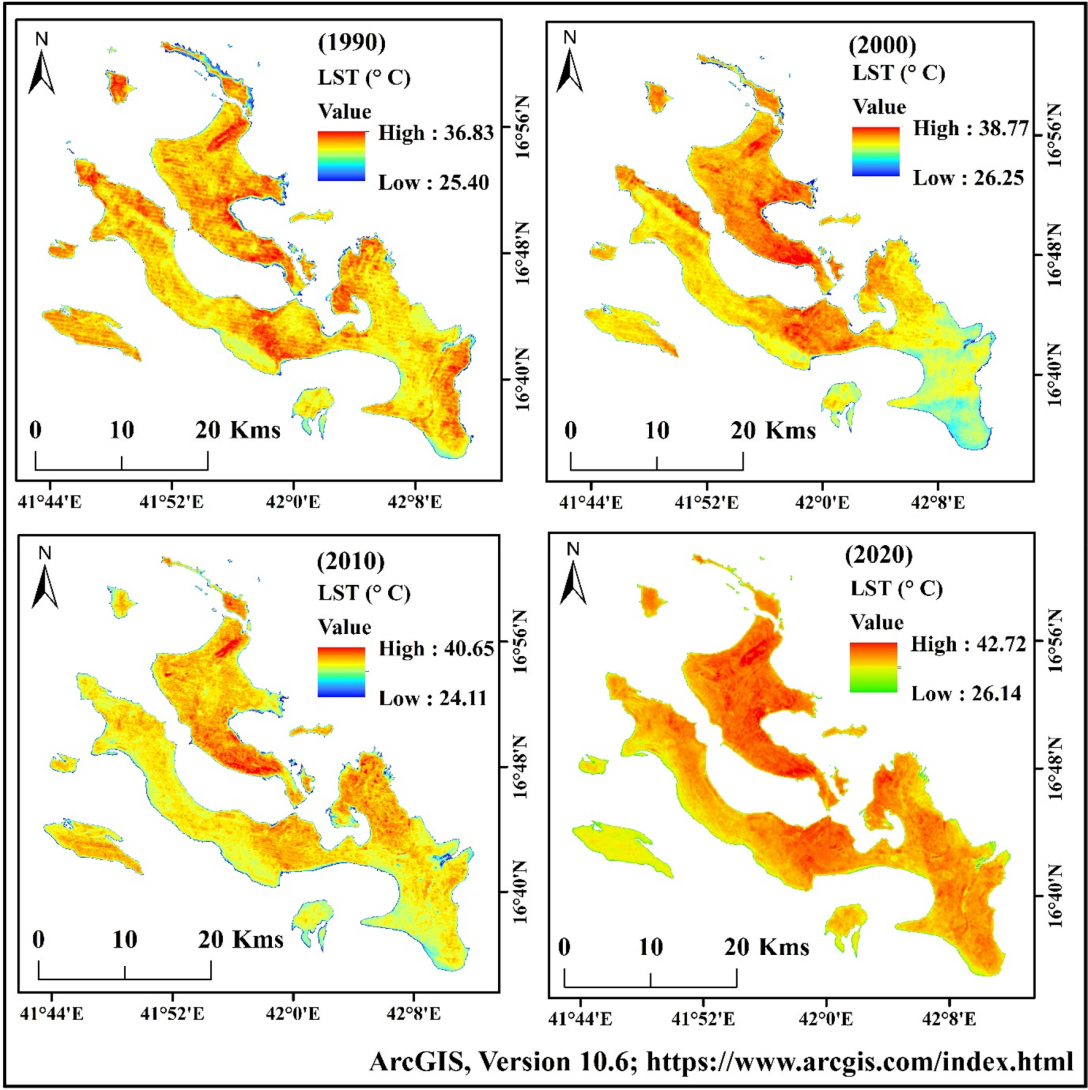
Land surface temperature (LST) maps of Farasan Islands for 1990, 2000, 2010, and 2020^31,64^. The satellite data was downloaded from USGS earth explorer (https://earthexplorer.usgs.gov/). The map was generated using ArcGIS software, version 10.6 (https://support.esri.com/zh-cn/products/desktop/arcgis-desktop/arcmap/10-6-1).

Satellite data for 4 years were used for calculating the surface temperature of Farasan Island, which were 1990, 2000, 2010 and 2020. The Landsat 5 TM and 8 OLI/TIRS data were used for this study. The highest temperatures in different years were 36.83 °C (1990), 38.77 °C (2000), 40.65 °C (2010) and 42.72 °C (2020) (Fig. 5)^31,64^, Figure generated using ArcGIS software 10.6. The lowest temperatures were 25.40 °C (1990), 25.25 °C (2000), 24.11 °C (2010), and 26.14 °C (2020). The annual temperature showed an increase of 0.196 °C/year. The highly influenced areas were the island's north, central, northwest, southwest and northeast parts. The temperature index clearly showed global climate change and urbanization impacts on the surface temperature of the island.

### Geospatial indices identification

The geospatial indices like NDVI and NDBI were used in this study to identify the vegetation and built-up scenario of Farasan Island. Vegetation is an important parameter for an environment that influences green space, maintains the balance of oxygen levels, provides thermal comfort, and reduces soil erosion. The built-up index was used for identifying the built-up scenarios of Farasan Island. The built-up index indicated that the scenarios were not similar for the initial phase of the study area. Urban expansion, population pressure and infrastructural development caused an increase in the built-up index values. The built-up index showed that 0.18 was the highest value in the initial phase, while the lowest was − 0.39 in 1990. After that, the built-up expansion increases the values of NDBI in the area.

**Figure 6:**
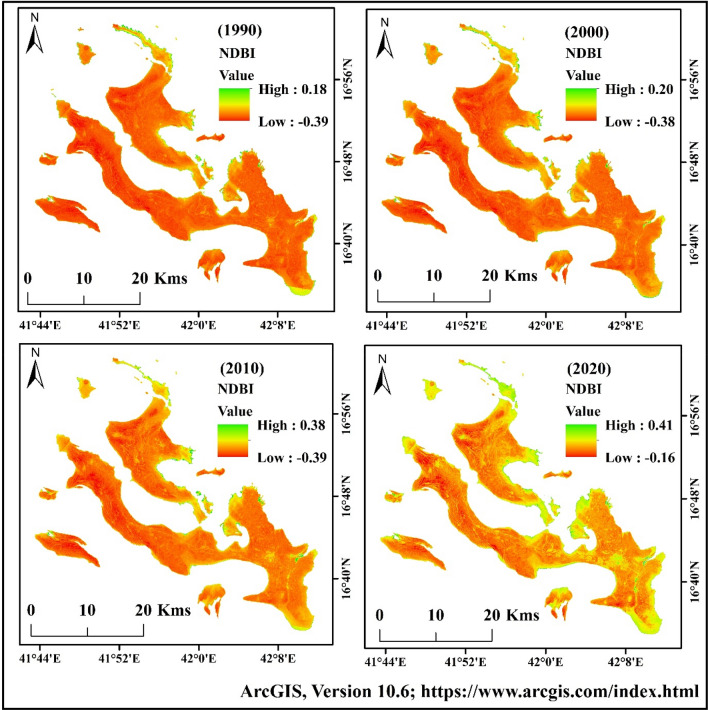
Normalized Different Built-up Index (NDBI) maps of Farasan Island for 1990, 2000, 2010, and 2020^31,64^. The satellite data was downloaded from USGS earth explorer (https://earthexplorer.usgs.gov/). The map was generated using ArcGIS software, version 10.6 (https://support.esri.com/zh-cn/products/desktop/arcgis-desktop/arcmap/10-6-1).

**Figure 7:**
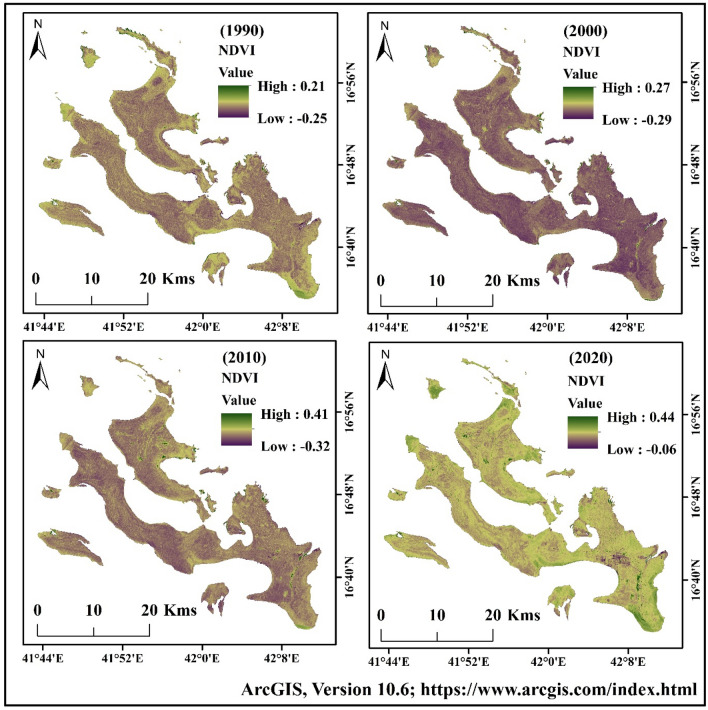
Normalized Different Vegetation Index (NDVI) maps of Farasan Island for 1990, 2000, 2010, and 2020^31,64^. The satellite data was downloaded from USGS earth explorer (https://earthexplorer.usgs.gov/). The map was generated using ArcGIS software, version 10.6 (https://support.esri.com/zh-cn/products/desktop/arcgis-desktop/arcmap/10-6-1).

The highest values of NDBI were 0.18 (1990), 0.20 (2000), 0.38 (2010) and 0.41 (2020), respectively (Fig. 6)^31,64^, Figure generated using ArcGIS software 10.6. Similarly, the NDVI maps indicated that the vegetation areas increased consistently. Vegetation in the lower and central parts of the area showed a higher increase. The NDVI values in different years were 0.21 (1990), 0.27 (2000), 0.41 (2010), and 0.44 (2020), respectively (Fig. 7)^31,64^, Figure generated using ArcGIS software 10.6. Due to urban expansion and reduction of the coastal region, the planners planted the vegetation in Farasan Island. The vegetation covers are more in the southern, central and northern parts. Besides, Al Meharrq, Qandal Forest, Khutob and the northern coastal side of this study area have more vegetation. Farasan, Sair, Abu Twoq, Khutob, Qummah, Al Qessar and Al Meharrg areas were dominated by built-up land, which also increased gradually.

### Shoreline change analysis

Anthropogenic activities, extreme environmental conditions, cyclones, sea-level rise, and tidal effects change the shoreline. The shoreline is important for detecting coastal vulnerability and monitoring global climate change. The present study showed that the shoreline of Farasan Island eroded in some parts and sediment deposition caused accretion in some parts. However, erosion was higher compared to accretion. The upper part of Abu Twoq was mainly eroded due to sea-level rise. Farasan Island experienced a huge amount of sediment deposition. Maximum sediments deposited into the Red Sea region like north of Jizan in the Al-Shuqaiq province since wadis which was conveyed to the South part of the wadis. The wind engendered the surface water currents transportation and scatter erogenous substantial particularly the micas to deep waters areas. Longshore areas also mean transportations of the sediments lengthways the coast consequential in the comprehensive shelf located in the southern parts of the Red Sea. Throughout sediment transportation, erogenous resources are conveyed to the East by the tidal currents. This results in sediment deposition on the beach area. The LST and the SST were also the influencing factors for coastal vulnerability, which affected local flora and fauna vulnerability. Besides, Humr, Qummah and Sajid areas were often eroded. The mangrove forest and the natural barriers protected the Island in many locations. However, sea-level rise destroyed the mangrove in some parts and made them vulnerable to erosion.

**Figure 8:**
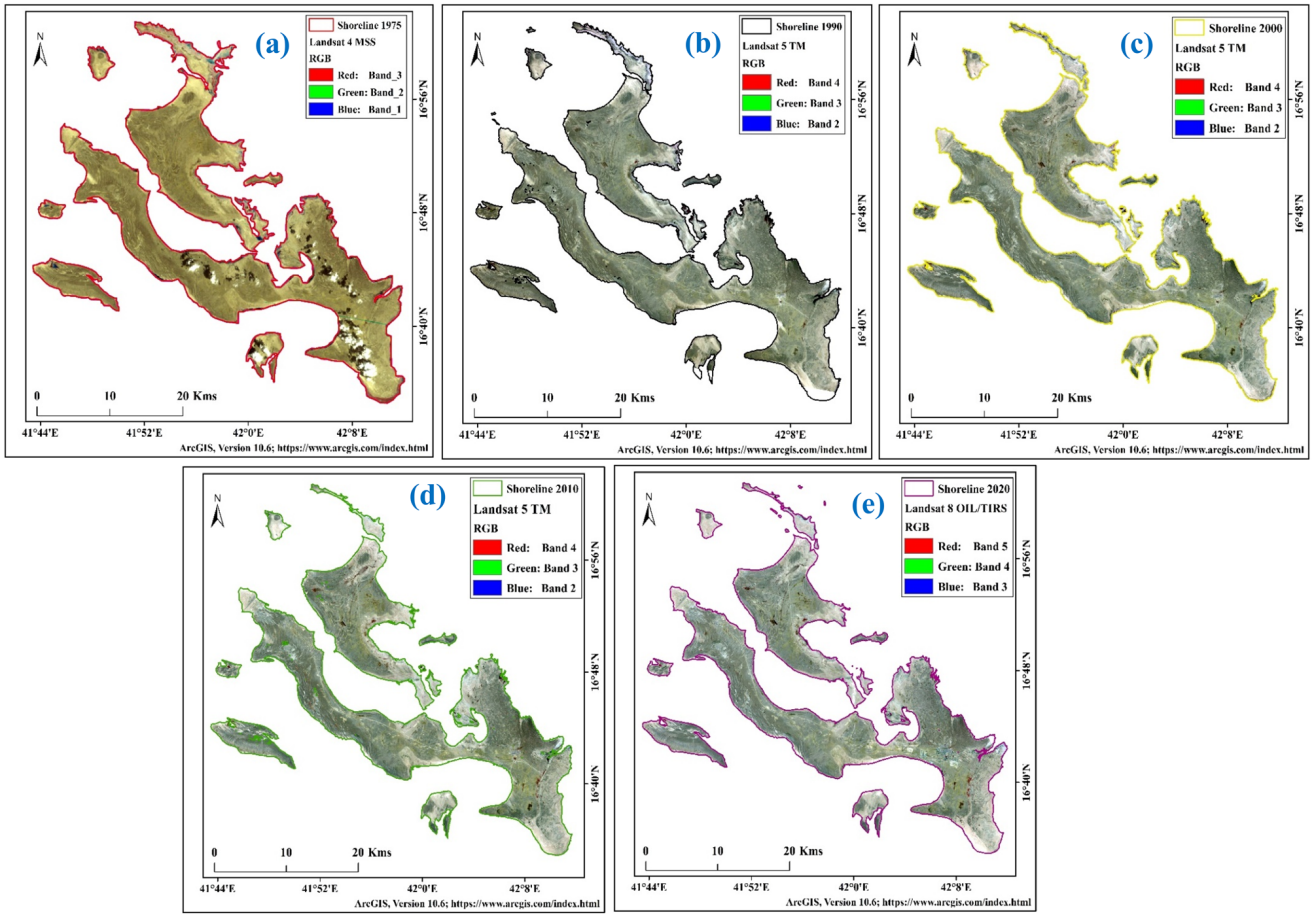
Shoreline of Farasan Islands in different years; (**a**) 1975; (**b**) 1990; (**c**) 2000; (**d**) 2010; and (**e**) 2020^31,64^. The satellite data was downloaded from USGS earth explorer (https://earthexplorer.usgs.gov/). The map was generated using ArcGIS software, version 10.6 (https://support.esri.com/zh-cn/products/desktop/arcgis-desktop/arcmap/10-6-1).

**Figure 9:**
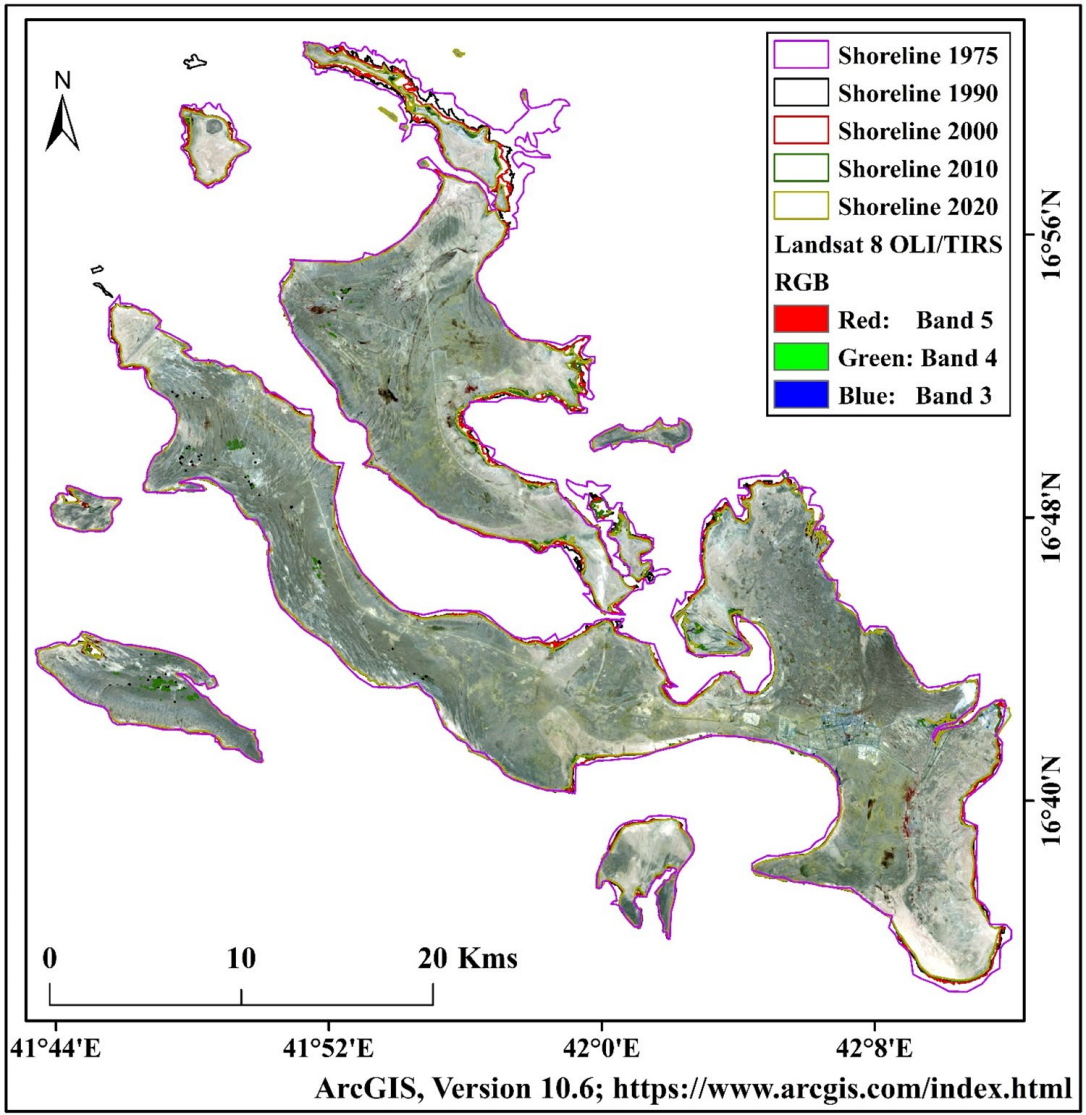
Graphical summary of the shoreline changes analysis maps of the Farasan Islands for different periods^31,64^. The satellite data was downloaded from USGS earth explorer (https://earthexplorer.usgs.gov/). The map was generated using ArcGIS software, version 10.6 (https://support.esri.com/zh-cn/products/desktop/arcgis-desktop/arcmap/10-6-1).

**Figure 10:**
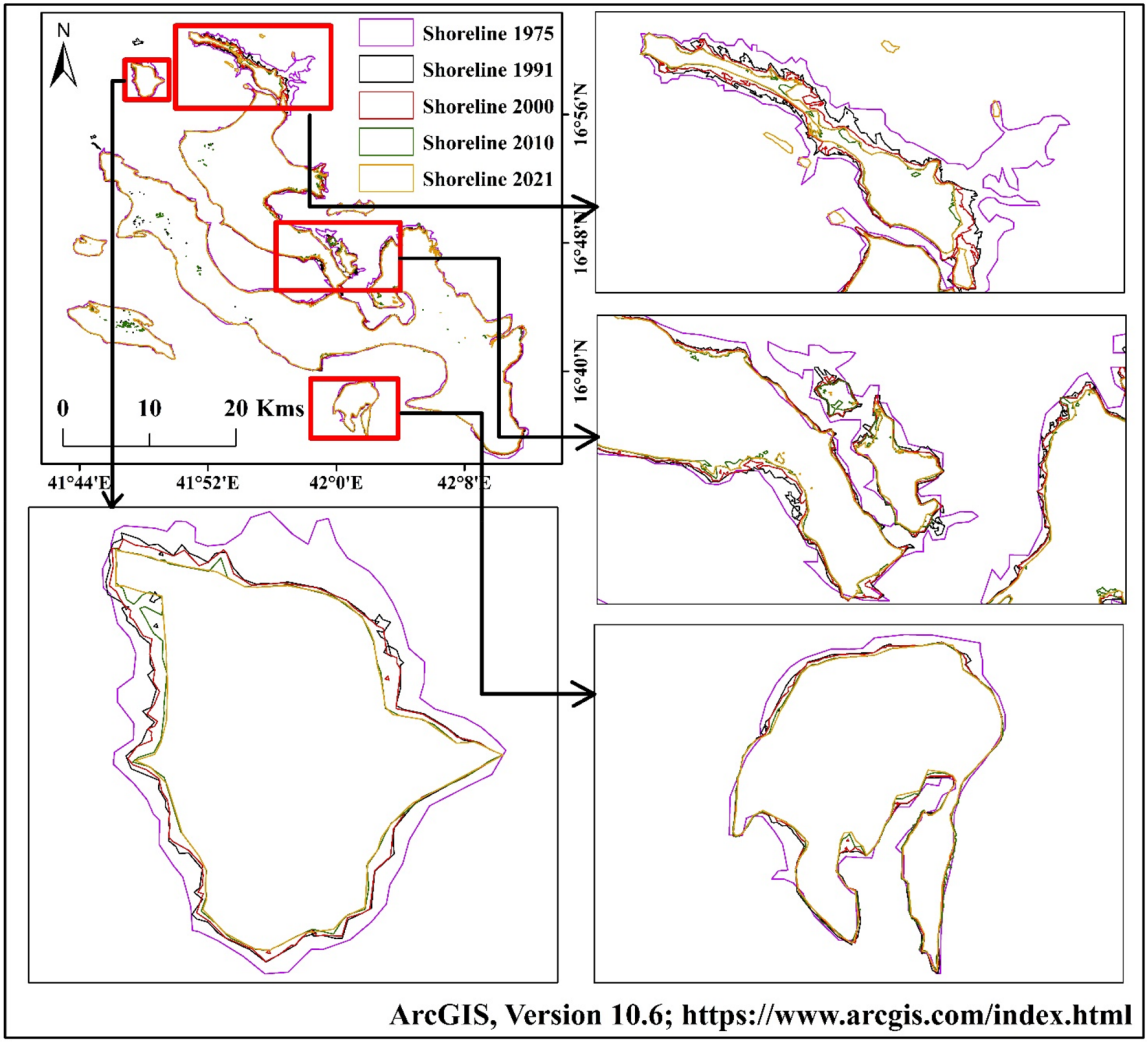
Shoreline shifting of Farasan Islands^31,64^. The satellite data was downloaded from USGS earth explorer (https://earthexplorer.usgs.gov/). The map was generated using ArcGIS software, version 10.6 (https://support.esri.com/zh-cn/products/desktop/arcgis-desktop/arcmap/10-6-1).

The total island areas were 695.22 km^2^ (1975), 645.75 km^2^ (1990), 635.34 km^2^ (2000), 617.93 km^2^ (2010), and 614.17 km^2^ (2020) respectively (Table 9). The upper part of Farasan Island was highly eroded. The upper island was connected with the main island, but after the sea level rose, the area was eroded and submerged in the sea (Fig. 8a–e)^31,64^, Figures generated using ArcGIS software 10.6. Forty-five years of shorelines were put in the same frame to locate the areal change of the Farasan Island (Fig. 9)^31,64^, Figure generated using ArcGIS software 10.6. The shoreline change analysis identified the shoreline shifting (Fig. 10)^31,64^, Figure generated using ArcGIS software 10.6. Tidal effect, sea-level rise and global climate change influenced the shoreline and its erosion and accretion.

**Table 9:** Total shoreline and erosional and accretional areas in different time scales.

Total area		Erosion/accretion area				
Year	Area (sq. km)	Year	Erosion (sq. km)	Accretion (sq. km)	Change (sq. km)	Remarks
1975	695.22	1975–1990	54.18	4.66	− 49.52	Erosion
1990	645.75	1990–2000	10.97	1.03	− 9.94	Erosion
2000	635.34	2000–2010	19.23	1.19	− 18.04	Erosion
2010	617.93	2010–2020	6.46	2.37	− 4.09	Erosion
2020	614.17	1975–2020	83.99	3.13	− 80.86	Erosion

**Figure 11:**
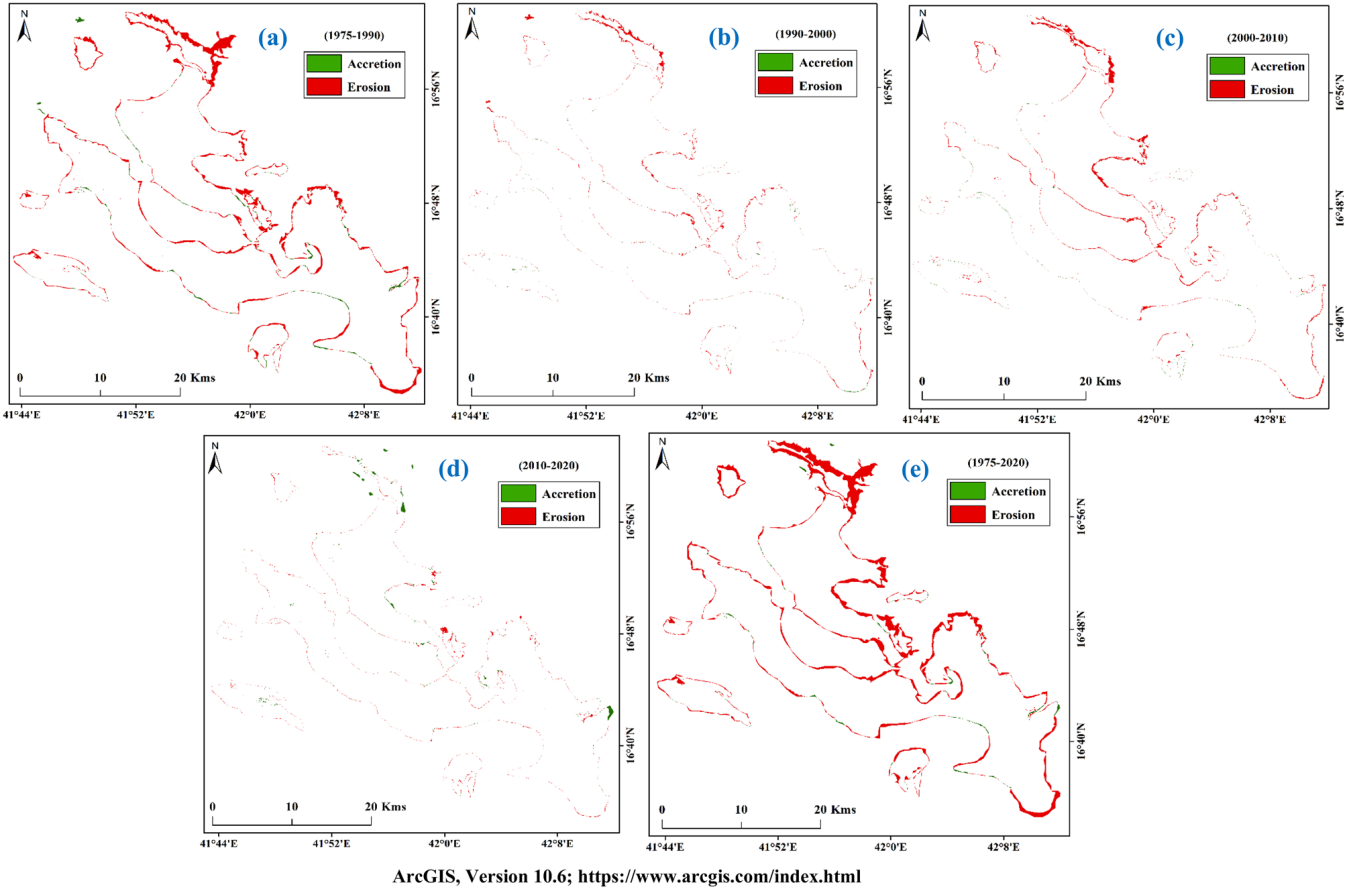
Erosion and accretion in Farasan Islands; (**a**) 1975–1990; (**b**) 1990–2000; (**c**) 2000–2010; (**d**) 2010–2020; and (**e**) 1975–2020^31,64^. The satellite data was downloaded from USGS earth explorer (https://earthexplorer.usgs.gov/). The map was generated using ArcGIS software, version 10.6 (https://support.esri.com/zh-cn/products/desktop/arcgis-desktop/arcmap/10-6-1).

Land transformation is a common phenomenon in coastal areas that has been accelerated due to global climate change and human activities. The SLR of the Red Sea has triggered the island's coastal vulnerability, erosion and accretion. Five types of maps were created to understand erosion and accretion areas (Fig. 11a–e)^31,64^, Figures generated using ArcGIS software 10.6. During 1975–1990, 54.18 km^2^ of shoreline was shifted (eroded), mostly in the north, north-east, east, and southwest, while 4.66 km^2^ of the area was gained (Table 9). Erosion and accretions were 10.97 km^2^ and 1.03 km^2^ respectively during 1990–2000, while 19.23 km^2^ and 1.19 km^2^ respectively during 2000–2010. The low shoreline shift was during 2010–2020, with 6.46 km^2^ erosion and 2.37 km^2^ accretion. The entire study area was eroded around 83.99 km^2^ and deposited 3.13 km^2^ during 1975–2020. The change analysis maps showed many changes in Farasan Island. The eroded area was 49.52 km^2^ (1975–1990), 9.94 km^2^ (1990–2000), 18.04 km^2^ (2000–2010), 4.09 km^2^ (2010–2020), and 80.86 km^2^ (1975–2020). Erosional activities in coastal are the most effective phenomenon. The global sea-level rise has triggered this activity.

### Impact of shoreline change

The Farasan Island experienced huge shoreline shifting in the last few decades due to sea-level rise in the Rea Sea. The analysis of shoreline shifting in Farasan Island showed a loss of around 81.05 km^2^ of land. Losses in mangrove forests were common in the coastal lowland area, mostly located in the upper part of Humr, near Sajid agglomeration, Qandal Forest, and Kaira area. Global climate change and sea levels have increased the vulnerability of the island. If this condition continues, a large portion of Farasan Island will be eroded.

## Discussion

The historical shoreline change is the most important research topic for generating, mapping and monitoring the global sea-level rise and land transformation. Landsat imagery is widely used for generating the high water level area and shoreline shift in the coastal regions. The geomorphological changes are also necessary for identifying coastal management. The present study revealed that sea-level rise and shoreline change in the last decades had influenced Farasan Island. SLR is the most important to identify the shoreline change in the coastal region, where ice melting, climate change, and vegetation damage increased the SLR and the coastal vulnerability and coastal erosion. The erosion rate of Farasan Island gradually increased due to the SLR. Therefore, SLR is one of the triggering factors for shoreline change.

Several circular to elliptical diapirs with diameters ranging between 3 and 35 km underlie an uplifted coral reef deposit in the northwest and southeast Farasan Islands^65^. These diapirs cause significant land deformations on the islands^66^. No study has been conducted to assess the role of salt diapirs on shoreline changes on Farahsan Island. A single study conducted on the evaluation of the salt-related land deformation in Jazan city diapir nearly 50 km east of Farahsan island by Pankratz et al.^67^ revealed large rates of land deformation in the Jazan diapir. The study showed a rise in diapir's elevation in the center up to 4.7 mm/year while subsiding the low-relief flats by − 7.5 mm/year. Although the land deformations due to diapirs are localized, they can affect the shoreline changes in the islands. The present study estimated the shoreline positions from the satellite imageries and extracted the changes due to land deformation by diapirs.

The natural resources of the island are very diverse. It has a high economic and recreation value^68^. Ecologically, it is rich with diverse species which need conservation^67–71^. The area is also highly perspective of culture and heritage. A recent study highlighted the significance of Farasan Island for heritage^72^. Therefore, it is important to understand the changes in the islands due to human activities.

The present study revealed that the islands' total land area was 695.22 km^2^ in 1975, which was reduced to 614.17 km^2^ in 2020. Landsat-8 data for geological mapping of the island were used^73^. The authors estimated the island's total area is 739 km^2^, which is very near to that estimated in this study. Another study was conducted for geomorphological mapping using multi-proxy data^74^. They reported depressions (lowlands) located on plateau surfaces as one of the major formations on the island. The present study also found lowlands as one of the major features of the island. Another study reported an increase in vegetation on the islands^75^. The findings of the present study also collaborate with it. Therefore, it can be remarked the satellite image-based shoreline estimation method used in this study is an effective tool for mapping erosion and accretion in remote and not easily accessible regions like Farasan Island.

The present study revealed both erosion and accretion of the Farasan Islands. There was a spatial variability in accretion and erosion. Overall, 83.99 km^2^ of land was lost while 3.13 km^2^ was gained during 1975–2020. This indicates a loss of nearly 80 km^2^ of land over the study period. Higher loss of land is due to unsustainable human activities and sea-level rise. Due to the SRL, Farasan Island faces huge land alteration along with shoreline mitigation. Also, increased human habits caused land alteration along with the thermal variation in the study area. Climate change is the triggering factor for SLR and human intervention the land alteration like vegetation degradation and built-up expansion. Vegetation degradation also is a triggering factor for shoreline change. Coastal erosion is reduced due to vegetation, but anthropogenic activities reduce the vegetation on Island, causing increased shoreline change. The shoreline was a noticeable change during the study period from 1975, but the anthropogenic activities stated in the most recent time. Therefore anthropogenic activities are not the only triggering factor for shoreline change. Climate change-induced SRL is also a main major cause of shoreline change.

The population of Farasan has increased significantly in the last decades. Economic activities are also accelerated in the region. The government planned many development activities in the islands, namely terminal, marina, hotel, cultural and environment administration, and retail. Besides, a harbour is planned to connect the island with the other globally important cities in the region. Increased population and development activities have made this ecologically rich region vulnerable^76^. However, some studies show no changes in some species. For example, Hausmann and Meredith-Williams^30^ showed no change in shell middens distribution on the island despite adverse climatic conditions. Another investigation reported a decrease in mountain gazelle in some islands in the Farasan^76^.

Besides human interventions, rising sea levels due to global warming also affected the Farasan Islands. The fluvial deposits from the wadis during the Holocene period formed the Red Sea coastal plain in the Farasan region. An examination of the island's geological structure using existing well log data^66^ revealed the stratigraphic succession of the islands as sand, coral reef limestone, marly limestone, shale, and evaporite, from top to bottom. The sandy deposition at the top has made the island more vulnerable to sea-level rise.

The surface elevation of the Red Sea is significantly influenced by water balance in winter. Therefore, it has significant seasonal variability in sea level. Global climate change has made the situation more severe. Besides, there is a unidirectional increase in sea level. Alawad et al.^77^ reported an increasing trend in the sea level of the Red Sea by about 0.28 cm/year during 1993–2010, which was very near to the global average. The present study revealed that increased sea level might be another cause of decreasing area of the Farasan Islands. An analysis of the trends in extreme waves near the shore showed an increase in the 95th and 99th percentile extreme waves^78^. The increase in extreme waves can also cause increased erosion in the islands.

## Conclusion

Shoreline shift analysis is the key to understand the erosion and accretion in the coastal areas. Global sea-level rise, extreme environmental events, and anthropogenic activities have triggered coastal vulnerability, influencing human habited, ecological diversity, and environmental conditions. Farasan Island is located in the southern part of the Red Sea, around 40 km from Jazan city. It is the habitat of many species and mangroves. Therefore, it is important from an ecological point of view. Sea-level rise and sediment dynamics caused a shift in the island's shoreline. The present study revealed that climate change had accelerated shoreline shift, which influenced the local environmental condition, misbalances of biodiversity and areal change. The study revealed a spatial variability of erosion and accretion, which caused erosion of 83.99 km^2^ of the land while accretion of only 3.13 km^2^ during 1975–2020. This indicates a loss of nearly 80 km^2^ of the island's land over the study period. Nearly 1975–1990, 54.18 km^2^ of the shoreline was shifted (eroded) during 1975–1990, mostly in the northeast, north, east, and southwest. In contrast, only 4.66 km^2^ of the area was increased through accretion. Erosions and accretions were 10.97 km^2^ and 1.03 km^2^ during 1990–2000, although 19.23 km^2^ and 1.19 km^2^ during 2000–2010. The least shift in shoreline was during 2010–2020, with 2.37 km^2^ accretion and 6.46 km^2^ erosion. The shoreline shifting was more prominent in the study area's northeast, central and southern parts. Shoreline change information provided in this paper can be helpful for the research community in understanding coastal vulnerability and global climate change-related information. Decision-makers, administrative departments, and other stakeholders can adopt strategies for coastal management and sustainable development in Farasan Island based on the study's findings. Further analysis can be conducted in the future to quantify the effects of different factors on shoreline changes. A hydro-dynamic model can be used to assess the changes in sea waves due to global warming and their impacts on accretion and erosion.

## Data Availability

The data used in the research modelling are freely available satellite data mentioned within the manuscript.
